# Serum and salivary inflammatory biomarkers in juvenile idiopathic arthritis—an explorative cross-sectional study

**DOI:** 10.1186/s12969-024-00972-6

**Published:** 2024-03-09

**Authors:** Lena Cetrelli, Anette Lundestad, Elisabet G. Gil, Johannes Fischer, Josefine Halbig, Paula Frid, Oskar Angenete, Annika Rosén, Karin B. Tylleskär, Keijo Luukko, Ellen Nordal, Anne N. Åstrøm, Marit S. Skeie, Astrid Kamilla Stunes, Athanasia Bletsa, Abhijit Sen, Astrid J. Feuerherm, Marite Rygg

**Affiliations:** 1 Center for Oral Health Services and Research (TkMidt), Trondheim, Norway; 2 Department of Clinical and Molecular Medicine, Faculty of Medicine and Health Sciences, Norwegian University of Science and Technology (NTNU), Trondheim, Norway; 3 Children’s Clinic, St. Olavs University Hospital, Trondheim, Norway; 4 Knarvik Orthodontics, Alver, Norway; 5 Department of Clinical Dentistry, The Faculty of Medicine, University of Bergen (UiB), Bergen, Norway; 6 Oral Health Centre of Expertise in Western Norway (TkV), Bergen, Norway; 7 Public Dental Health Service Competence Centre of Northern Norway (TkNN), Tromsø, Norway; 8 Department of Clinical Dentistry, The Arctic University of Norway (UiT), Tromsø, Norway; 9 Department of Otorhinolaryngology, Division of Oral and Maxillofacial Surgery, University Hospital North Norway, Tromsø, Norway; 10 Department of Radiology and Nuclear Medicine, St. Olav Hospital HF, Trondheim University Hospital, Trondheim, Norway; 11 Department of Circulation and Medical Imaging, Faculty of Medicine and Health Sciences, Norwegian University of Science and Technology (NTNU), Trondheim, Norway; 12 Department of Oral and Maxillofacial Surgery, Haukeland University Hospital, Bergen, Norway; 13 Child and Youth Clinic, Haukeland University Hospital, Bergen, Norway; 14 Department of Clinical Medicine, The Arctic University of Norway (UiT), Tromsø, Norway; 15 Department of Pediatrics, University Hospital of Northern Norway, Tromsø, Norway; 16 Department of Oral and Maxillofacial Surgery, Eastman Institute, Public Dental Health Service, Stockholm, Sweden; 17 Department of Public Health and Nursing, Faculty of Medicine and Health Sciences, Norwegian University of Science and Technology (NTNU), Trondheim, Norway

**Keywords:** Juvenile idiopathic arthritis, Children, Adolescents, Biomarkers, Inflammation, Serum, Saliva, Disease activity

## Abstract

**Background:**

Biomarkers may be useful in monitoring disease activity in juvenile idiopathic arthritis (JIA). With new treatment options and treatment goals in JIA, there is an urgent need for more sensitive and responsive biomarkers.

**Objective:**

We aimed to investigate the patterns of 92 inflammation-related biomarkers in serum and saliva in a group of Norwegian children and adolescents with JIA and controls and in active and inactive JIA. In addition, we explored whether treatment with tumor necrosis factor inhibitors (TNFi) affected the biomarker levels.

**Methods:**

This explorative, cross-sectional study comprised a subset of children and adolescents with non-systemic JIA and matched controls from the Norwegian juvenile idiopathic arthritis study (NorJIA Study). The JIA group included individuals with clinically active or inactive JIA. Serum and unstimulated saliva were analyzed using a multiplex assay of 92 inflammation-related biomarkers. Welch’s t-test and Mann–Whitney U-test were used to analyze the differences in biomarker levels between JIA and controls and between active and inactive disease.

**Results:**

We included 42 participants with JIA and 30 controls, predominantly females, with a median age of 14 years. Of the 92 biomarkers, 87 were detected in serum, 73 in saliva, and 71 in both biofluids. A pronounced difference between serum and salivary biomarker patterns was found. Most biomarkers had higher levels in serum and lower levels in saliva in JIA versus controls, and in active versus inactive disease. In serum, TNF and S100A12 levels were notably higher in JIA and active disease. The TNF increase was less pronounced when excluding TNFi-treated individuals. In saliva, several biomarkers from the chemokine family were distinctly lower in the JIA group, and levels were even lower in active disease.

**Conclusion:**

In this explorative study, the serum and salivary biomarker patterns differed markedly, suggesting that saliva may not be a suitable substitute for serum when assessing systemic inflammation in JIA. Increased TNF levels in serum may not be a reliable biomarker for inflammatory activity in TNFi-treated children and adolescents with JIA. The lower levels of chemokines in saliva in JIA compared to controls and in active compared to inactive disease, warrant further investigation.

**Supplementary Information:**

The online version contains supplementary material available at 10.1186/s12969-024-00972-6.

## Background

Juvenile idiopathic arthritis (JIA) is the most common chronic rheumatic condition affecting children before the age of 16 years [1–3]. JIA can cause severe joint damage and compromise skeletal growth and functional ability. The clinical course and pattern of joint inflammation in JIA vary between disease categories and fluctuate over time. The pathogenesis of JIA is not fully understood, but a combination of genetic predisposition and environmental and immunological factors have been proposed [4]. The current knowledge indicates a complex interaction between various immune cells and proinflammatory molecules which triggers the immunopathological process causing the joint inflammation and tissue damage characteristic of JIA [4]. Pro-inflammatory biomarkers appear to be dominating in active JIA, while anti-inflammatory and regulatory biomarkers are more common during remission [5]. Systemic JIA has been shown to have a different pathogenesis and biomarker profile than that of other JIA categories [5, 6]. Increased levels of several proinflammatory biomarkers have been found in circulation and the synovial fluid of affected joints in individuals with JIA [5]. The two pro-inflammatory biomarkers, tumor necrosis factor alpha (from here on referred to as TNF) and interleukin 6 (IL6) are important therapeutic targets in the treatment of JIA [7–9]. High levels of the calcium-binding proteins S100A12 and S100A8/A9 have been associated with inflammatory activity, and prediction of treatment response and flare in JIA, however, study results are inconsistent [10–13]. Composite measures consisting of both clinical and biochemical markers have been used to describe and quantify disease activity in JIA [14–16].

In the last decades, the introduction of biologic disease-modifying anti-rheumatic drugs (bDMARDs), with TNF inhibitors (TNFi) most used, has dramatically improved the prognosis in JIA [17]. Current treatment of JIA aims to induce clinical or even biochemical remission as soon as possible using individualized approaches in a “treat to target” strategy [18]. However, despite more effective drugs, treatment-resistant disease and disease flares are still a problem for many children [19, 20]. Furthermore, low levels of subclinical inflammation may persist in clinically inactive JIA without being detected [11, 21]. Thus, highly sensitive and responsive biomarkers are demanded to monitor disease activity and identify any remaining inflammation and risk of flare.

Traditionally, measuring and monitoring biomarker levels have been performed in serum. Blood sampling is an invasive method that may cause pain and discomfort and can be challenging in children. The collection of saliva, on the other hand, is simple, minimally invasive, and causes little discomfort. As saliva contains many of the inflammatory molecules traditionally measured in serum, it may to some extent reflect an individual’s systemic health status [22]. Studies on inflammatory biomarkers in the saliva of children and adolescents with JIA are limited [23, 24] and therefore, further investigation is needed.

We aimed to investigate the patterns of a large set of inflammation-related biomarkers in serum and saliva in a group of Norwegian children and adolescents with JIA and controls, and in active and inactive JIA. In addition, we explored the influence of TNFi treatment on biomarker levels.

## Methods

### Study design and setting

Our study sample consisted of a selected subset of children and adolescents with active and inactive non-systemic JIA and controls from the NorJIA study. The NorJIA study is a Norwegian, longitudinal, multicenter cohort study of 4–16-year-old children with JIA and controls, taking place from 2015–2020 (ClinicalTrials.gov, No: NCT03904459). The children with JIA were recruited from outpatient Pediatric Rheumatology clinics at the University Hospital of Northern Norway in Tromsø, St. Olavs University Hospital in Trondheim, and Haukeland University Hospital in Bergen. The control group consisting of age- and sex-matched children and adolescents without JIA, were recruited from nearby public dental service clinics, as part of the regular oral health examinations of Norwegian children aged 3–18 years. Recruitment of controls was organized by the Public Dental Health Service Competence Centre of Northern Norway (TkNN) in Tromsø, the Center of Oral Health Services and Research (TkMidt) in Trondheim, and the Oral Health Centre of Expertise in Western Norway (TkV) in Bergen. Children with JIA were examined by pediatric rheumatologists at the three study centers, and extensive clinical, anthropometric, and demographic data were registered. Imaging, patient-reported questionnaires, and blood and saliva sampling were also included. The NorJIA study has been described in detail [25, 26].

In the current study, the inclusion criteria for the JIA group were active, or inactive disease. The controls were matched on age and sex, and they should not have JIA or other rheumatic diseases. All study participants should have both medical and oral health examinations and serum and saliva samples available for biomarker assessment. For both groups, children with any major medical conditions such as cancer, hereditary deformities of the face, skeletal dysplasia, or not having signed an informed consent were excluded.

### Data collection and measures

Demographic and anthropometric data collection included age, sex, weight, height, and parental education level. Co-existing somatic health conditions and medication were registered for the JIA group and the controls. Clinical disease-related data included age at disease onset, disease duration, JIA category according to the International League of Associations for Rheumatology (ILAR) classification criteria [2], the physician’s global assessment of disease activity scored on a 21-numbered circle visual analog scale (PhysGA VAS), the number of active joints, disease activity, and ongoing medication with disease-modifying anti-rheumatic drugs (DMARDs). Body mass index (BMI) was calculated (weight [kg]/(height [meters(m)])^2^). Adjustment for age and sex resulted in iso-BMI groups corresponding to adult BMI groups, including overweight/obesity ≥ 25 kg/m^2^ according to The International Obesity Task Force recommendations [27, 28]. To assess disease activity, the variables *active* and *inactive* JIA according to Wallace and the American College of Rheumatology (ACR 2011) endorsed criteria [15, 16] were used. Active disease was defined as continuous activity since disease onset or flare. Inactive disease was defined as no active arthritis, no fever, rash, serositis, splenomegaly, or generalized lymphadenopathy due to JIA, no active uveitis, and normal CRP and/or ESR, morning stiffness below 15 min, and the PhysGA VAS = 0, whether the patient was on or off medication and independent of the duration of the inactive state. The Juvenile Idiopathic Arthritis Disease Activity Score based on 71 joints (JADAS71), a validated composite score measuring disease activity, was also assessed [14]. This scoring tool has a scoring range of 0–101, where 0 = no activity, and 101 = maximum disease activity. The items included in the JADAS71 are the physician’s global assessment of disease activity VAS (0–10), the parents/patient global assessment of disease impact on wellbeing VAS (0–10), the number of active joints (0–71), and the normalized erythrocyte sedimentation rate (ESR) (0–10).

### Serum and salivary samples

At the study visit, which took place throughout the day (between 09–15 a.m.), a standardized set of blood tests was collected by venipuncture, including CRP and ESR, from all participants with JIA. Given their approval, blood tests were also collected and analyzed from the controls. Additional serum was aliquoted and stored at -80 °C until further analysis.

A standardized method for collecting, storing, and handling the saliva samples was used. Unstimulated whole saliva was collected by the drooling method [29]. All saliva samples were to be collected between 09.00—12.00 a.m. The participants were instructed not to eat, drink, rinse their mouths, or brush their teeth for two hours before the sampling, nor should they take any other medication than prescribed. The unstimulated salivation rate in mL/minute was calculated by dividing the collected volume of saliva by the time used (6 min). Homogenization by pipetting was performed before the saliva was aliquoted, frozen at the latest 30 min after sampling, and stored at -80 °C until analyzed. For the current sub-study, one serum and saliva sample was thawed once, and 50μl of each sample was added to 96 well PCR plates and shipped on dry ice to Olink Bioscience in Uppsala, Sweden for biomarker measurements [30].

### Measurements of inflammatory biomarkers

Inflammatory biomarkers in serum and saliva were assessed by the Proseek Multiplex proximity enhanced extension assay (PEA) [30]. The Olink Target 96 inflammation panel included 92 high-quality assays for biomarkers related to inflammation, including groups of chemokines, chemokine receptors, cytokines, cytokine receptors, enzymes, and growth factors. The average intra-assay coefficient of variability (%CV) was 3% for serum and 4% for saliva. Data was expressed as normalized protein expression (NPX) values, an arbitrary unit in a Log2 scale. In contrast to accurate biomarker concentrations, NPX values are relative quantification values. A difference in one NPX corresponds to a doubling of the protein concentration. Biomarkers with more than 60% of the values below the level of detection (LOD) were excluded from further analyses.

### Statistical analysis

Descriptive statistics were used for demographic, anthropometric, and clinical characteristics of JIA and controls, and disease-related characteristics for the JIA group. Data are presented as frequency (percentages) for categorical variables, mean and standard deviation (± SD) for normally distributed continuous variables, and median and interquartile range (IQR) for skewed data. For all serum and salivary inflammatory biomarkers, the mean NPX values (± SD) were used when comparing JIA and controls, and children with active and inactive JIA, respectively.

Normality was assessed by the Shapiro–Wilk test and visual inspection of quantile–quantile (Q-Q) plots. A Welch’s two-sample t-test was used to compare the statistical differences in mean NPX values of normally distributed biomarkers between JIA versus controls, and active versus inactive JIA [31]. The Mann–Whitney U-test was used for the skewed data. From all biomarkers, we chose to select those that a) were significantly different (*p* < 0.05), or b) had a difference between mean NPX values of at least 0.4 when comparing JIA versus controls, and active versus inactive disease. The cut-off value of 0.4 was chosen as a suitable difference to capture biomarkers of potential clinical significance that might be suitable candidates for future studies.

Forest plots illustrating the differences between mean NPX values with 95% confidence intervals for the selected biomarkers in the two comparison groups are presented.

To explore the influence of TNFi treatment on biomarker levels in serum and saliva, we performed sub-analyses excluding children treated with TNFi from the analyses.

Statistical analyses were performed using the IBM Statistical Package for Social Sciences version 29 (SPSS Inc., Illinois, USA).

## Results

In this study, we included 42 children and adolescents with non-systemic JIA, selected to represent both clinically active and inactive disease, and 30 age- and sex-matched controls without JIA (Table 1). All non-systemic JIA categories, apart from polyarticular RF positive JIA, were represented (Table 2). The JIA group included 21 individuals with active and 21 with inactive disease, of which 12 had been inactive for more than twelve months without any medication and thus had reached a state of remission off medication. The mean JADAS71 score in the active group was 8.72 (± SD 5.55) and 1.00 (± SD 1.64) in the inactive group. In the total JIA group, 24 were on DMARDs, including eight on synthetic DMARDs (sDMARDs) alone (all of these using methotrexate) and 16 on biologic DMARDs (bDMARDs), of which 14 were on TNFi. Two children treated with synthetic and biologic DMARDs also used steroids. Of the remaining 18 children not taking any DMARDs, five were only taking NSAIDs and 13 did not take any medication at the time of sampling.

**Table 1:** Characteristics of the study groups

Characteristics	JIA (*N* = 42)	Controls (*N* = 30)
Age at study visit, median years (IQR)	13.9 (11.3–15.1)	14.3 (12.2–16.4)
Sex, females, n (%)	29 (69.0)	22 (73.3)
Iso-BMI > 25^a^, n (%)	10 (23.8)	6 (20.0)
Parental education^b^, high level, n (%)	19 (50.0)	17 (65.4)
CRP ≥ 5 mg/L^c^, n (%)	3 (7.1)	3 (10.3)
ESR > 10 mm/h^d^, n (%)	16 (38.1)	4 (13.8)
Salivary flow rate^e^, mL/min, mean (SD)	0.4 (± 0.5)	0.6 (± 0.6)

*JIA* Juvenile idiopathic arthritis, *IQR* Interquartile range, *CRP* C-reactive protein in milligram per liter plasma or serum, *SD* standard deviation, *ESR* Erythrocyte sedimentation rate measured in millimeter per hour

^a^iso-BMI = body mass index adjusted for age and sex, corresponding to adult BMI groups according to The International Obesity Task Force for overweight/obesity: iso-BMI ≥ 25 kg/m2

^b^Parental education level, mother and father, the parent with the highest level determined the grouping: Low = primary and high school (**≤ **13 years of education), High = university-level education

^c^CRP ≥ 5 indicates active inflammation, CRP missing in 1 in the control group

^d^ESR > 10 mm/h indicates active inflammation, ESR missing in *n* = 1 in the control group

^e^Unstimulated salivation: Saliva collected by drooling and spitting into a collection tube. Participants were instructed not to eat, drink, brush their teeth, or take any other than prescribed medication before collecting saliva, *n* = 4 saliva samples from the JIA group are missing

**Table 2:** Characteristics of the juvenile idiopathic arthritis group (*n* = 42)

Clinical characteristics	Value
Age at study visit, years, median (IQR)	13.9 (11.3–15.1)
Age at disease onset, years, median (IQR)	8.4 (2.7–11.1)
Disease duration, years, median (IQR)	4.8 (2.8–8.3)
JIA category^a^, n (%)	
Oligoarticular persistent	15 (35.7)
Oligoarticular extended	3 (7.1)
Polyarticular RF negative	10 (23.8)
Psoriatic	2 (4.8)
Enthesitis-related	8 (19.0)
Undifferentiated	4 (9.5)
ANA positive^b^, n (%)	14 (35.0)
HLA-B27 positive, n (%)	13 (31.0)
Disease activity^c^, n (%)	
Remission off medication	12 (28.6)
Inactive	9 (21.4)
Active	21 (50.0)
JADAS71^d^ > 0, n (%)	28 (70.0)
PhysGA VAS > 0, n (%)	14 (33.3)
Number of individuals with active joints^e^, n (%)	9 (21.4)
DMARDs^f^ ongoing, n (%)	24 (57.1)
Steroids ongoing, n (%)	2 (4.7)
NSAIDs ongoing, n (%)	15 (35.7)
No medication, n (%)	13 (31.0)

*JIA* juvenile idiopathic arthritis, *IQR* inter-quartile range, *ANA* antinuclear antibodies, *RF* Rheumatoid factor, *HLA-B27* human leukocyte antigen B27, *JADAS71* Juvenile Idiopathic Arthritis Activity Score based on 71 joints, *VAS* visual analog scale, *PhysGA VAS* physician’s global assessment of disease activity using a 21 numbered circle VAS (0 = no activity, 10 = maximum activity), *DMARDs* disease-modifying anti-rheumatic drugs, *bDMARDs* biologic DMARDs, *NSAIDs* non-steroid anti-inflammatory drugs

^a^According to the International League of Association for Rheumatology (ILAR) classification criteria. Only non-systemic JIA categories were included. No children had RF positive polyarticular JIA

^b^ ANA positive is defined as two positive ANA tests with HEp-2 substrate measured at least 3 months apart

^c^ Disease status according to Wallace et al. Remission off medication = inactive disease off medication for ≥ 12 months. Inactive = inactive disease on medication for < 6 months, or off medication < 12 months, or remission on medication (inactive disease on medication for ≥ 6 months). Active = continuous active disease or flare

^d^ JADAS71 is a composite scoring tool measuring disease activity (range 0–101 0 = no activity, 101 = maximum activity), consisting of the physician’s global assessment of disease activity VAS (0–10), the parents/patient global assessment of disease impact on wellbeing VAS (0–110), the number of active joints (0–71), and the normalized erythrocyte sedimentation rate (ESR) (0–10). (2 missing)

^e^ Number of participants with physician-determined swollen joints or joints with loss of range of motion and joint pain or tenderness

^**f**^ Either synthetic (methotrexate and mycophenolate mofetil) and/or biologic (etanercept, infliximab, adalimumab, certolizumab, tocilizumab, abatacept) DMARDs

Other medication not related to JIA and comorbidities in the study groups are listed in Supplemental Table S1.

### Differences in serum and salivary biomarker levels in JIA compared to controls

**Fig. 1:**
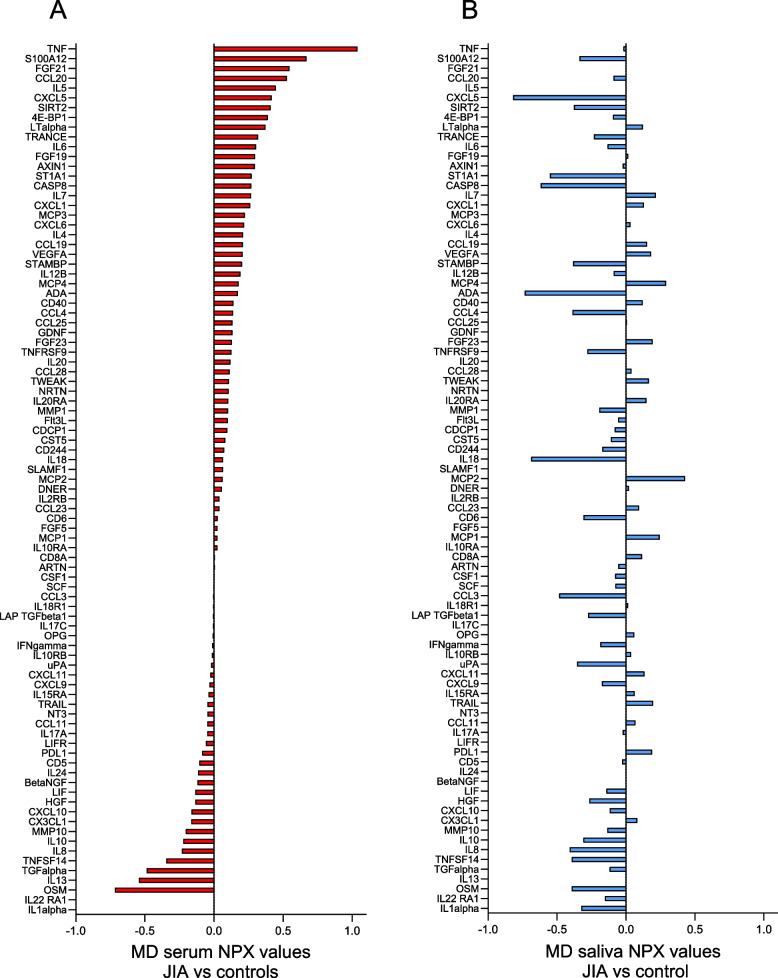
Visual illustration of the differences between mean NPX values of serum and salivary biomarkers in children with JIA (*N* = 42) compared to controls (*N* = 30). MD = mean difference, NPX = normalized protein expression value, an arbitrary unit on a Log2 scale according to the Proseek multiplex-enhanced extension assay provided by Olink Proteomics. The serum biomarkers in Panel A (red bars) are sorted in descending order and the salivary biomarkers in Panel B (blue bars) are sorted according to the serum biomarkers. Positive differences indicate higher and negative differences indicate lower biomarker levels in JIA compared to controls. The total number of serum samples in JIA was 42 and in controls 29 (1 missing), and saliva samples in JIA were 38 (4 missing), and 30 in controls. The biomarkers with NPX values below the level of detection in more than 60% of the samples have been excluded (5 biomarkers in serum and 19 in saliva)

After excluding biomarkers with levels below the LOD in more than 60% of the samples (5 in serum and 19 in saliva) (Supplemental Table S2), 87 and 73 biomarkers were detected in serum and saliva, respectively. Of these, 71 biomarkers were found in both serum and saliva, 16 were unique for serum, and two for saliva (Supplemental Table S3 and S4). The differences between mean NPX values in JIA compared to controls of all detected biomarkers in serum sorted in descending order, with the corresponding biomarker differences found in saliva, are shown in Fig. 1. Overall, the biomarker levels were different in serum and saliva. In serum, 57/87 (65%) of the biomarkers had higher levels in JIA, as opposed to saliva where 44/73 (60%) of the biomarkers had lower levels in JIA compared to controls. Eighteen biomarkers had higher and 14 had lower levels in JIA both in serum and saliva. The rest of the biomarkers were oppositely expressed in the two biofluids. An overview of all biomarkers with mean NPX values (± SD) in serum and saliva in the JIA group and controls is presented in Supplemental Table S3.

**Fig. 2:**
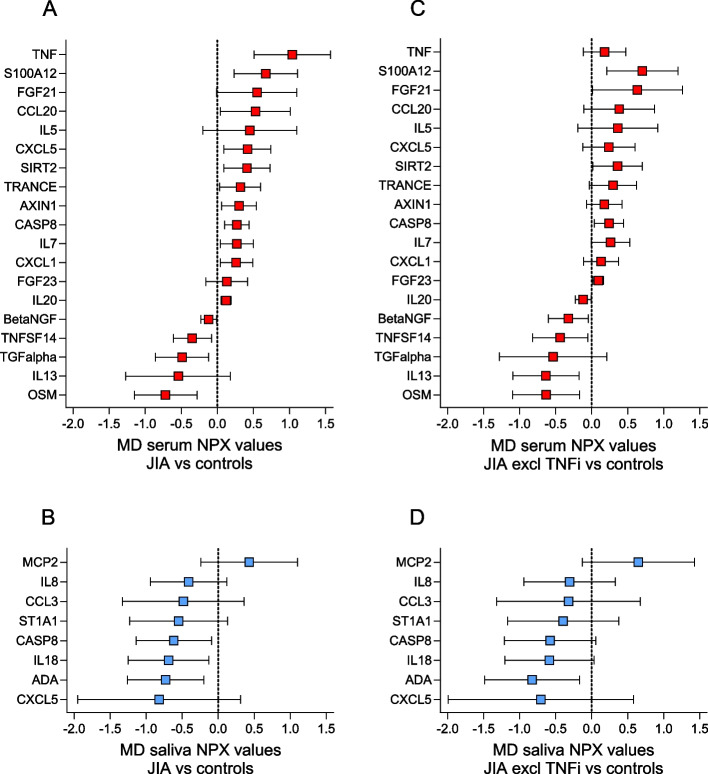
Differences between mean NPX values with 95% confidence intervals of selected serum and salivary biomarkers in children with JIA (*n* = 42) compared to controls (*n* = 30). NPX = normalized protein expression. MD = mean difference. Biomarkers were selected if the difference between mean NPX values was significantly different (*p* < 0.05) or had a difference of at least 0.4 when comparing children with JIA with controls. Positive differences indicate higher and negative differences indicate lower biomarker levels in JIA compared to controls. The selected serum biomarkers (*n* = 18, red bars) and salivary biomarkers (*n* = 8, blue bars) were sorted in descending order. Panels A and B show the difference in serum (A) and saliva (B) for the total group of children with JIA (*n* = 42) compared to controls (*n* = 30), while panels C (serum) and D (saliva) show the same differences in *n* = 28 children with JIA after exclusion of 14 children treated with TNF inhibitors

In total, 18 biomarkers in serum and 8 in saliva met our cut-off criteria by either having a significant difference or a difference in mean NPX values of at least 0.4 when comparing JIA and controls (Fig. 2, Panels A and B). Of the serum biomarkers with higher levels in JIA compared to controls (positive NPX values), TNF had the most pronounced difference in mean NPX value of 1.04, followed by S100A12, fibroblast growth factor 21 (FGF21), and CC motif chemokine ligand 20 (CCL20) with differences of more than 0.5. Of the biomarkers with lower levels in JIA compared to controls (negative NPX values), oncostatin M (OSM), and transforming growth factor alpha (TGFalpha) had the most pronounced mean NPX differences.

In saliva, all the selected salivary biomarkers had lower levels in JIA compared to controls, except monocyte chemotactic protein 2 (MCP2). CXC motif chemokine ligand 5 (CXCL5), adenosine deaminase (ADA), IL18, and caspase-8 (CASP8) had the most pronounced differences in NPX values. CXCL5 and CASP8 were the only selected biomarkers detected both in serum and saliva. However, both were oppositely expressed.

### Differences in serum and salivary biomarker levels in active compared to inactive JIA

**Fig. 3:**
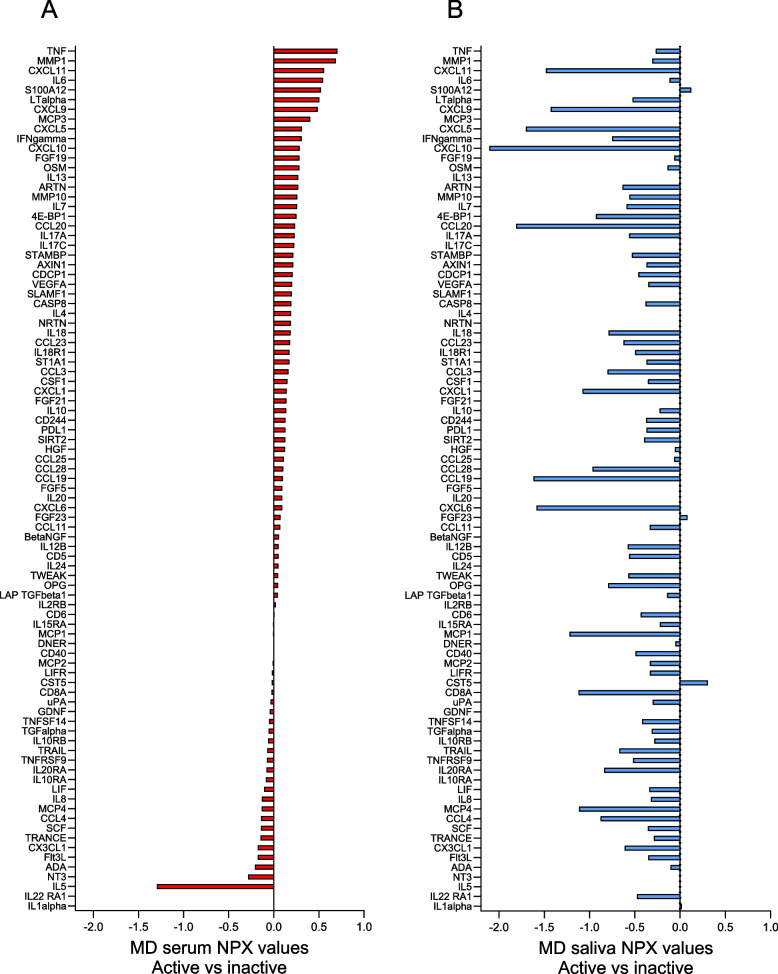
Visual illustration of the differences between mean NPX values of serum and salivary biomarkers in individuals with active (*n* = 21) compared to inactive JIA (*n* = 21). MD = mean difference, NPX = normalized protein expression value, an arbitrary unit on a Log2 scale according to the Proseek multiplex-enhanced extension assay provided by Olink Proteomics. The serum biomarkers in Panel A (red bars) are sorted in descending order and the salivary biomarkers in Panel B (blue bars) are sorted according to the serum biomarkers. Positive differences indicate higher and negative differences indicate lower biomarker levels in active compared to inactive JIA. The total number of serum samples in active and inactive JIA was 21, respectively, and saliva samples were 20 in active, and 18 in inactive JIA (4 missing). The biomarkers with NPX values below the level of detection in more than 60% of the samples have been excluded (5 biomarkers in serum and 19 in saliva)

When comparing active and inactive JIA, the difference in biomarker levels between serum and saliva was even more pronounced (Fig. 3). In serum, 70% of the biomarkers had higher levels, while 95% of the biomarkers in saliva had lower levels in active compared to inactive JIA. Two biomarkers (S100A12 and FGF23) had higher levels in active compared to inactive disease both in serum and saliva, and 19 had lower levels in both biofluids. An overview of all the biomarkers with mean NPX values (± SD) in serum and saliva in active and inactive JIA is presented in Supplemental Table S4.

**Fig. 4:**
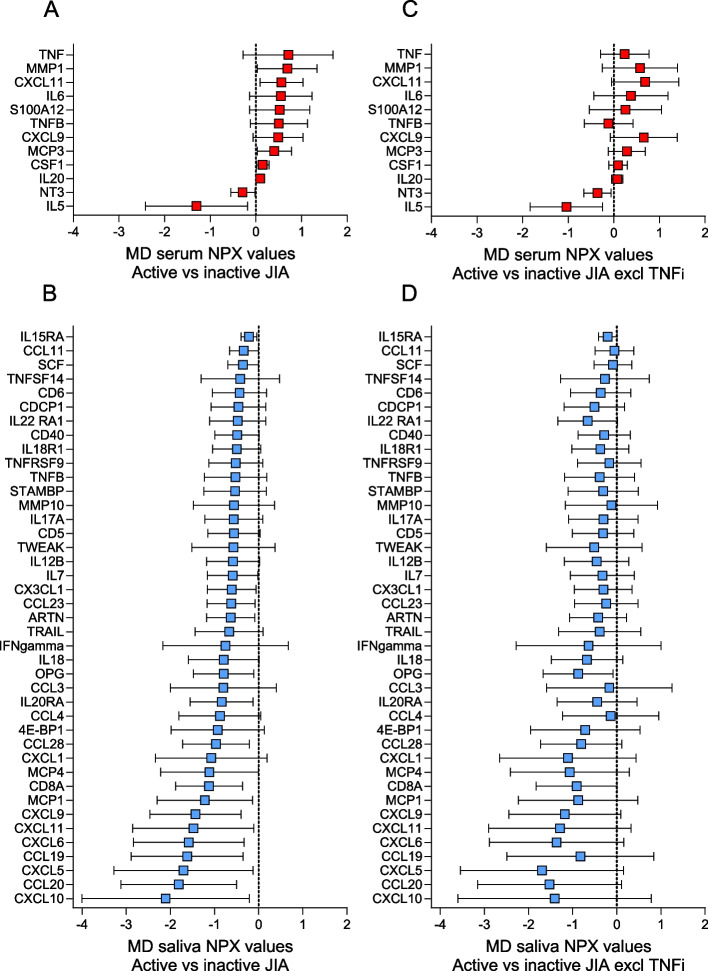
Differences between mean NPX values with 95% confidence intervals of serum and salivary biomarkers in individuals with active (*n* = 21) compared to inactive JIA (*n* = 21). NPX = normalized protein expression. MD = mean difference. Biomarkers were selected if the difference between NPX values was significantly different (*p* < 0.05) or had a difference of at least 0.4 NPX when comparing children with active and inactive JIA. Positive differences indicate higher and negative differences indicate lower biomarker levels in active compared to inactive JIA. The selected serum biomarkers (*n* = 12, red bars) and salivary biomarkers (*n* = 41, blue bars) were sorted in descending order. Panels A and B show the difference in serum (**A**) and saliva (**B**) for the total group av children with active (*n* = 21) compared to inactive JIA (*n* = 21), while Panels C (serum) and D (saliva) show the same differences in *n* = 28 children with JIA after exclusion of 14 children treated with TNF inhibitors

Of the selected biomarkers meeting our cut-off criteria for differences between mean NPX values, 12 were detected in serum and 41 in saliva (Fig. 4, Panels A and B). Among the selected biomarkers with higher levels in active compared to inactive JIA in serum, five had an NPX difference of more than 0.5. Of these, TNF had the most pronounced difference with a mean NPX value of 0.71, followed by matrix metalloproteinase 1 (MMP1), CXCL11, IL6, and S100A12.

In saliva, all the selected biomarkers had lower levels in active compared to inactive JIA. Twelve of these biomarkers had a difference in mean NPX value of more than 1, and one biomarker more than 2. CXCL11 had the most pronounced difference, followed by CCL20, CXCL5, CCL19, CXCl6, CXCl11, CXCL9, Monocyte chemoattractant protein-1 (MCP1), T-cell surface glycoprotein *CD8* alpha chain (CD8A), MCP4, CXCL1 and CCL28 with differences in mean NPX value of more than 1 (Fig. 4, Panel B). Of the selected biomarkers, CXCL11 and CXCL9 were present in both serum and saliva, however oppositely expressed.

### Influence of TNFi on biomarker levels in serum and saliva

TNF was the biomarker with the most pronounced difference in mean NPX values in serum when comparing JIA with controls, and active with inactive disease. When we excluded the samples from the 14 TNFi-treated individuals from the analysis, the difference in TNF levels between JIA and controls changed from 1.04 (95% CI, 0.51 to 1.57) before exclusion (JIA, *n* = 42) to 0.18 (95% CI, -0.12 to 0.47) after exclusion (JIA, *n* = 28) (Fig. 2, A and C). Apart from TNF, there were only minor differences observed among the other selected serum biomarkers after the exclusion of TNFi-treated individuals. A similar observation was made when comparing differences in mean NPX values after excluding TNFi-treated individuals in active compared to inactive disease. The difference in TNF levels changed from 0.71 (95% CI, -0.28 to 1.69) to 0.24 (95% CI, -0.29 to 0.77) (Fig. 4, Panels A and C).

We found no substantial changes among the selected salivary biomarkers when TNFi-treated individuals were excluded neither in JIA versus controls nor in active versus inactive JIA (Fig. 2, Panels B and D, and Fig. 4, Panels B and D).

## Discussion

In this explorative study of Norwegian children and adolescents with JIA and controls, the levels of 92 inflammation-related biomarkers in serum and saliva were investigated. We found markedly different biomarker patterns in serum and saliva. Although 71% of the biomarkers were found in both biofluids, most biomarkers were oppositely expressed, with higher levels in serum and lower levels in saliva in JIA versus controls, and active versus inactive JIA. We confirmed higher serum levels of TNF, IL6, and S100A12, biomarkers known to be increased in JIA. In saliva, several biomarkers from the chemokine subfamilies had lower levels in JIA compared to controls. This was even more pronounced in active compared to inactive JIA. The distinctly higher TNF levels in serum found in the JIA group and in active disease were markedly reduced in sub-analyses excluding TNFi-treated children.

To the best of our knowledge, there are no previous studies of children and adolescents with non-systemic JIA assessing such high numbers of inflammation-related biomarkers in both serum and saliva. Most studies investigating biomarker levels in JIA focus on a limited number of selected biomarkers in blood or synovial fluid and they rarely include matched salivary biomarkers [5, 10, 12, 32–36]. This makes comparisons with other studies difficult.

Similar to our study, higher levels of several pro-inflammatory biomarkers in blood have been found in JIA compared to controls, and in active compared to inactive JIA, including TNF, IL6, and S100A12 [5, 10, 12, 32–35]. However, no difference or lower levels of these biomarkers have also been reported [33, 34, 36]. Differences in study design, analytic methods, or heterogeneities of study groups might explain these inconsistencies.

Several biomarkers in serum and plasma including TNF, IL6, and S100A12 have been associated with high disease activity, and higher risk of persistent disease or flare [10, 12, 32, 34]. This is consistent with our results of higher biomarker levels in serum in active compared to inactive JIA. To our knowledge, several of the selected biomarkers in active compared to inactive JIA in our study, such as CXCL11, lymphotoxin alpha (LT-alpha, formerly referred to as TNF-beta), and IL20, have not previously been targets for further investigations in serum in young individuals with non-systemic JIA.

The number of studies using the same multiplex assay as in our study to assess levels of either serum or salivary biomarkers in JIA compared to controls is scarce. However, the same assay was used in a recent prospective study of biomarkers in plasma in newly diagnosed treatment-naïve children with active non-systemic JIA and controls [10]. In line with our study, most of the 92 inflammation-related biomarkers, including IL6 and S100A12, had higher levels in JIA compared to controls. Further comparisons are difficult as they had a different study design and did not include measurements of salivary biomarkers. The same multiplex assay was used to compare levels of serum and salivary biomarkers in adults with active inflammatory bowel disease (IBD) and controls [37]. Direct comparisons with this study cannot be made, however, overall, and in line with our results, most serum biomarkers differed from the corresponding salivary biomarkers.

Previous studies have demonstrated increased serum levels of TNF in TNFi-treated individuals [32–35, 38]. This increase has been explained by circulating TNF binding to the TNFi molecule, creating inactive but measurable complexes [35, 38]. Thus, the high level of TNF detected in our study might be due to TNFi treatment, rather than a sign of ongoing disease activity. This indicates that increased serum levels of TNF in TNFi-treated individuals may not be a reliable biomarker for disease activity in JIA.

Most studies of saliva in young individuals with JIA focus on oral health. Studies investigating the relation between inflammatory biomarkers in saliva and disease-related characteristics in JIA are scarce. A recent study assessing the levels of 21 inflammatory biomarkers in stimulated saliva, found no difference in biomarker levels between children with active JIA and controls [23]. In the current study, we found lower levels of several of these 21 biomarkers in unstimulated saliva (TNF, CCL3, CCL11, IL8, IL10, and CXCL9) in JIA compared to controls, and in active compared to inactive JIA. However, the two studies are not comparable due to different immunoassays and study design.

We found that most of the salivary biomarkers had lower levels in JIA compared to controls, and when comparing individuals with active and inactive JIA, nearly all biomarkers were markedly lower in active JIA. Among the selected 41 salivary biomarkers, 16 biomarkers belonged to the chemokine sub-families. Of these, CXCL11 had the most pronounced difference in biomarker level. In contrast to our results, no differences in chemokine levels in stimulated saliva between children with active JIA and controls have been reported [23]. Chemokines are pro-inflammatory proteins that stimulate immune cell migration and are involved in immune and inflammatory responses [39]. Salivary flow rate has been shown to influence the cytokine levels in saliva in healthy children and adolescents, with levels decreasing with increased salivary flow rate [40]. Changes in salivary flow rate might have similar effects on chemokine levels. However, we found that even though the salivation rates tended to be lower in JIA versus controls, the levels of chemokines were lower in JIA versus controls, and in active versus inactive disease. Thus, it seems unlikely that salivary flow rate can explain the differences in chemokine levels in our study.

This study has several strengths. An extensive panel of inflammation-related biomarkers in both serum and saliva were investigated, and the study included age- and sex-matched controls. The JIA group was well characterized and experienced pediatric rheumatologists used validated measurements for disease activity. Apart from polyarticular RF positive JIA, the full spectrum of non-systemic JIA categories was represented, including balanced subgroups of children with clinically active and inactive disease. There are also limitations in this study. Our study was underpowered and therefore the results must be interpreted with caution. It was a cross-sectional study with only one-time measures of serum and salivary biomarkers. Blood samples were taken at the clinical examination in a non-fasting state which took place at different time points during a working day. Although we matched for age and sex, biomarker analyses in both serum and saliva might have been influenced by several other factors such as co-existing health conditions, medication, and diurnal and circadian variations [23, 40–45]. Oral health conditions and drugs not related to JIA, such as antihistamines, may have influenced biomarker levels in saliva [40, 46]. Furthermore, minor deviations from the standardized method for saliva handling, especially time for sampling and between sampling and freezing, might have affected the results.

## Conclusion

In this explorative study, the serum and salivary biomarker patterns differed markedly, suggesting that saliva may not be a suitable substitute for serum when assessing systemic inflammation in JIA. Increased TNF levels in serum may not be a reliable biomarker for inflammatory activity in TNFi-treated individuals with JIA. The markedly lower chemokine levels in saliva in JIA compared to controls and in active compared to inactive disease warrant further investigation.

### Supplementary Information

**Additional file 1: Supplemental Table S1.** Comorbidities and co-medication in the study groups. The table shows the number of individuals with co-existing health conditions and medication unrelated to juvenile idiopathic arthritis. It also shows the health conditions and medication of the control group.

**Additional file 2: Supplemental Table S2.** Inflammatory biomarkers excluded from analyses. The table shows serum and salivary biomarkers with normalized protein expression (NPX) values below the level of detection in more than 60% of the study sample.

**Additional file 3: Supplemental Table S3.** Serum and salivary inflammation-related biomarkers in JIA versus controls. The table shows all the detected biomarkers in serum and saliva, with mean NPX values and differences between mean NPX values in JIA versus controls.

**Additional file 4: Supplemental Table S4.** Serum and salivary inflammation-related biomarkers in active versus inactive JIA. The table shows all the detected biomarkers in serum and saliva, with mean NPX values and differences between mean NPX values in active versus inactive JIA.

## Data Availability

The dataset used in this study can be made available from the corresponding author at reasonable request.

## Abbreviations

JIA: Juvenile idiopathic arthritis
NorJIA study: Norwegian Juvenile Idiopathic Arthritis study
ILAR: International League of Associations for Rheumatology
DMARDs: Disease-modifying anti-rheumatic drugs
sDMARDs: Synthetic DMARDs
bDMARDs: Biologic DMARDs
TNFi: Tumor necrosis factor alpha inhibitor
PEA: Proximity enhanced extension assay
NPX: Normalized protein expression
%CV: Average intra-assay coefficient of variability
LOD: Level of detection
