# Supplementary material for: Serum and salivary inflammatory biomarkers in juvenile idiopathic arthritis—an explorative cross-sectional study
Source: Pediatr Rheumatol Online J. 2024 Mar 9;22:36. doi: 10.1186/s12969-024-00972-6 (PMC10924355; doi:10.1186/s12969-024-00972-6)
Supplement: Supplementary file 1 — Additional file 1: Supplemental Table S1. Comorbidities and co-medication in the study groups. The table shows the number of individuals with co-existing health conditions and medication unrelated to juvenile idiopathic arthritis. It also shows the health conditions and medication of the control group. [file 12969_2024_972_MOESM1_ESM.docx]

**Supplemental Table S1.** Comorbidities and co-medication in the study groups

|  | JIA, total  N=42 | Active^a^ JIA  N=21 | Inactive^a^ JIA  N=21 | | Controls  N=29 |
| --- | --- | --- | --- | --- | --- |
| **Comorbidities** | 7 | 3 | 4 | 3 | |
| Psoriasis | 1 |  | 1 |  | |
| Hypothyroidism | 1 |  | 1 |  | |
| Asthma | 4 | 2 | 2 | 1 | |
| Atopic eczema | 2 | 2 |  |  | |
| Allergies, unspecified | 20 | 8 | 12 | 8 | |
| ADHD | 1 |  |  | 1 | |
| Psychiatric disorder, unspecified | 1 |  |  | 1 | |
| **Medication** |  |  |  |  | |
| Levothyroxine | 1 |  | 1 |  | |
| Asthma medication, regular | 2 |  |  | 1 | |
| Antihistamines, regular | 1 |  |  |  | |
| ADHD medication, stimulants | 1 |  |  | 1 | |

JIA = Juvenile idiopathic arthritis, ADHD = attention deficit hyperactivity disorder. The table shows the number of individuals with other health conditions and medication unrelated to juvenile idiopathic arthritis. It also shows the health conditions of the control group.

^a^Disease status according to the definition by Wallace et al. Active disease was defined as continuous activity since disease onset or flare. Inactive disease was defined as no active arthritis, no fever, rash, serositis, splenomegaly, or generalized lymphadenopathy due to JIA, no active uveitis, and normal CRP and/or ESR, morning stiffness below 15 minutes, and the Physician’s global assessment of disease activity reported on a visual analogue scale = 0, whether the patient was on or off medication and independent of the duration of the inactive state.
