# Supplementary material for: Serum and salivary inflammatory biomarkers in juvenile idiopathic arthritis—an explorative cross-sectional study
Source: Pediatr Rheumatol Online J. 2024 Mar 9;22:36. doi: 10.1186/s12969-024-00972-6 (PMC10924355; doi:10.1186/s12969-024-00972-6)
Supplement: Supplementary file 2 — Additional file 2: Supplemental Table S2. Inflammatory biomarkers excluded from analyses. The table shows serum and salivary biomarkers with normalized protein expression (NPX) values below the level of detection in more than 60% of the study sample. [file 12969_2024_972_MOESM2_ESM.docx]

**Supplemental Table S2**. Inflammatory biomarkers excluded from analyses^a^

| **Serum biomarkers**  **(n = 5)** | **Salivary biomarkers**  **(n = 19)** |
| --- | --- |
| IL1alpha | MCP3 |
| IL2 | GDNF |
| TSLP | IL17C |
| IL22RA1 | IL2RB |
| IL33 | IL2 |
|  | TSLP |
|  | SLAMF1 |
|  | IL10RA |
|  | FGF5 |
|  | FGF21 |
|  | BetaNGF |
|  | IL24 |
|  | IL13 |
|  | IL20 |
|  | IL33 |
|  | IL4 |
|  | NRTN |
|  | NT3 |
|  | IL5 |

^a^ Biomarkers with normalized protein expression (NPX) values below the limit of detection in more

than 60% of the study sample of the 92 inflammatory proteins in the Olink inflammation panel used

in this study
