# Supplementary material for: Serum and salivary inflammatory biomarkers in juvenile idiopathic arthritis—an explorative cross-sectional study
Source: Pediatr Rheumatol Online J. 2024 Mar 9;22:36. doi: 10.1186/s12969-024-00972-6 (PMC10924355; doi:10.1186/s12969-024-00972-6)
Supplement: Supplementary file 3 — Additional file 3: Supplemental Table S3. Serum and salivary inflammation-related biomarkers in JIA versus controls. The table shows all the detected biomarkers in serum and saliva, with mean NPX values and differences between mean NPX values in JIA versus controls. [file 12969_2024_972_MOESM3_ESM.docx]

| **Supplemental Table S3.** Serum and salivary inflammation-related biomarkers in JIA versus controls | | | | | | | | | | |  | |  |
| --- | --- | --- | --- | --- | --- | --- | --- | --- | --- | --- | --- | --- | --- |
|  |  | **SERUM** |  |  | | |  | |  | **SALIVA** | |  | |
|  | **JIA** (n = 42) | **Controls** (n = 29) |  |  | | |  | **JIA** (n = 38) | | **Controls** (n = 30) | |  | |
| **Proteins** | **Mean NPX** | **Mean NPX** | **Diff. mean NPX^a^** |  |  | **Mean NPX** | | | | **Mean NPX** | | **Diff. mean NPX^a^** | |
|  | **(±SD)** | **(±SD)** |  |  |  | **(±SD)** | | | | **(±SD)** | |  |  |
| TNF | 5.70 (1.59) | 4.66 (0.50) | **1.04** |  |  | 2.57 (0.97) | | | | 2.59 (1.08) | | -0.02 | |
| S100A12 | 6.55 (1.07) | 5.88 (0.79) | **0.67** |  |  | 8.04 (1.13) | | | | 8.37 (0.78) | | -0.34 | |
| FGF21**^b^** | 4.85 (1.36) | 4.31 (1.00) | 0.55 |  |  |  | | | |  | |  | |
| CCL20 | 7.54 (0.83) | 7.01 (1.09) | **0.53** |  |  | 7.07 (2.21) | | | | 7.17 (2.07) | | -0.09 | |
| IL5**^b^** | 2.6 (1.86) | 2.15 (0.84) | 0.45 |  |  |  | | | |  | |  | |
| CXCL5 | 13.59 (0.65) | 13.18 (0.69) | **0.42** |  |  | 11.47 (2.46) | | | | 12.29 (2.18) | | -0.82 | |
| SIRT2 | 4.25 (0.72) | 3.84 (0.61) | **0.41** |  |  | 3.21 (1.00) | | | | 3.58 (1.00) | | -0.38 | |
| 4E-BP1 | 8.27 (0.82) | 7.88 (1.01) | 0.39 |  |  | 7.46 (1.66) | | | | 7.55 (1.25) | | -0.09 | |
| LT-alpha | 6.83 (1.01) | 6.46 (0.44) | 0.37 |  |  | 2.67 (1.11) | | | | 2.55 (0.97) | | 0.12 | |
| TRANCE | 6.90 (0.54) | 6.58 (0.62) | **0.32** |  |  | 2.96 (1.00) | | | | 3.19 (1.35) | | -0.23 | |
| AXIN1 | 3.08 (0.56) | 2.79 (0.46) | **0.30** |  |  | 1.77 (0.60) | | | | 1.80 (0.50) | | -0.02 | |
| FGF19 | 9.08 (0.87) | 8.78 (0.68) | 0.30 |  |  | 1.92 (0.55) | | | | 1.90 (0.73) | | 0.02 | |
| IL6 | 4.24 (1.11) | 3.93 (0.98) | 0.30 |  |  | 6.17 (1.59) | | | | 6.30 (1.37) | | -0.13 | |
| CASP8 | 2.65 (0.46) | 2.38 (0.27) | **0.27** |  |  | 6.04 (1.20) | | | | 6.65 (0.97) | | **-0.62** | |
| IL7 | 5.86 (0.51) | 5.59 (0.47) | **0.27** |  |  | 4.82 (0.91) | | | | 4.60 (0.85) | | 0.22 | |
| ST1A1 | 4.38 (0.74) | 4.10 (1.00) | 0.27 |  |  | 4.93 (1.39) | | | | 5.48 (1.39) | | -0.55 | |
| CXCL1 | 10.61 (0.43) | 10.35 (0.50) | **0.26** |  |  | 10.98 (1.99) | | | | 10.85 (1.86) | | 0.13 | |
| CXCL6 | 10.96 (0.57) | 10.74 (0.65) | 0.22 |  |  | 8.02 (2.07) | | | | 7.98 (1.84) | | 0.03 | |
| MCP3**^b^** | 3.53 (0.63) | 3.30 (0.63) | 0.22 |  |  |  | | | |  | |  | |
| CCL19 | 10.64 (0.76) | 10.43 (0.84) | 0.21 |  |  | 3.51 (2.10) | | | | 3.36 (1.57) | | 0.15 | |
| IL4**^b^** | 2.15 (1.09) | 1.94 (0.68) | 0.21 |  |  |  | | | |  | |  | |
| VEGFA | 12.09 (0.56) | 11.89 (0.39) | 0.21 |  |  | 13.97 (0.68) | | | | 13.79 (0.63) | | 0.18 | |
| STAMBP | 5.35 (0.51) | 5.14 (0.41) | 0.20 |  |  | 5.23 (1.11) | | | | 5.62 (1.04) | | -0.38 | |
| IL12B | 7.67 (0.47) | 7.48 (0.49) | 0.19 |  |  | 3.1 (0.94) | | | | 3.19 (1.12) | | -0.09 | |
| MCP4 | 15.07 (0.59) | 14.89 (0.58) | 0.18 |  |  | 5.57 (1.78) | | | | 5.27 (1.53) | | 0.29 | |
| ADA | 6.98 (0.42) | 6.81 (0.44) | 0.17 |  |  | 6.88 (1.32) | | | | 7.61 (0.86) | | **-0.73** | |
| CCL4 | 7.90 (0.58) | 7.76 (0.52) | 0.14 |  |  | 3.44 (1.49) | | | | 3.83 (1.59) | | -0.39 | |
| CD40 | 12.54 (0.29) | 12.4 (0.29) | 0.14 |  |  | 11.91 (0.80) | | | | 11.78 (0.95) | | 0.12 | |
| CCL25 | 6.99 (0.43) | 6.85 (0.51) | 0.13 |  |  | 1.46 (0.21) | | | | 1.45 (0.27) | | 0.01 | |
| FGF23 | 2.96 (0.58) | 2.83 (0.60) | 0.13 |  |  | 1.8 (0.58) | | | | 1.61 (0.37) | | 0.19 | |
| GDNF**^b^** | 3.13 (0.61) | 3.00 (0.48) | 0.13 |  |  |  | | | |  | |  | |
| TNFRSF9 | 8.87 (0.49) | 8.74 (0.56) | 0.13 |  |  | 5.17 (0.97) | | | | 5.45 (1.23) | | -0.28 | |
| IL20**^b^** | 1.45 (0.14) | 1.34 (0.14) | **0.12** |  |  |  | | | |  | |  | |
| CCL28 | 2.67 (0.46) | 2.56 (0.36) | 0.11 |  |  | 7.84 (1.25) | | | | 7.80 (1.01) | | 0.04 | |
| NRTN**^b^** | 1.65 (0.60) | 1.54 (0.29) | 0.11 |  |  |  | | | |  | |  | |
| TWEAK | 11.39 (0.30) | 11.28 (0.34) | 0.11 |  |  | 7.92 (1.46) | | | | 7.76 (1.22) | | 0.17 | |
| CDCP1 | 3.73 (0.37) | 3.63 (0.45) | 0.10 |  |  | 8.01 (0.97) | | | | 8.09 (0.93) | | -0.08 | |
| Flt3L | 10.12 (0.40) | 10.02 (0.44) | 0.10 |  |  | 4.23 (0.78) | | | | 4.28 (0.91) | | -0.06 | |
| IL20RA | 2.00 (0.59) | 1.90 (0.43) | 0.10 |  |  | 4.11 (1.13) | | | | 3.96 (0.84) | | 0.15 | |
| MMP1 | 11.22 (1.09) | 11.12 (0.94) | 0.10 |  |  | 6.49 (1.28) | | | | 6.69 (1.10) | | -0.19 | |
| CST5 | 6.34 (0.43) | 6.26 (0.52) | 0.08 |  |  | 8.61 (0.85) | | | | 8.72 (0.64) | | -0.11 | |
| CD244 | 8.18 (0.30) | 8.10 (0.42) | 0.07 |  |  | 2.29 (0.66) | | | | 2.46 (0.95) | | -0.17 | |
| IL18 | 9.51 (0.36) | 9.44 (0.42) | 0.07 |  |  | 9.96 (1.28) | | | | 10.64 (1.03) | | **-0.69** | |
| SLAMF1**^b^** | 2.65 (0.35) | 2.59 (0.290) | 0.07 |  |  |  | | | |  | |  | |
| DNER | 10.27 (0.25) | 10.21 (0.23) | 0.06 |  |  | 11.96 (0.25) | | | | 11.94 (0.21) | | 0.02 | |
| MCP2 | 10.59 (0.62) | 10.53 (0.86) | 0.06 |  |  | 4.01 (1.47) | | | | 3.58 (1.31) | | 0.43 | |
| CCL23 | 10.74 (0.39) | 10.7 (0.46) | 0.04 |  |  | 2.42 (0.89) | | | | 2.33 (0.88) | | 0.10 | |
| IL2RB**^b^** | 2.31 (0.32) | 2.27 (0.36) | 0.04 |  |  |  | | | |  | |  | |
| CD6 | 6.76 (0.37) | 6.74 (0.55) | 0.03 |  |  | 2.03 (0.96) | | | | 2.34 (1.19) | | -0.31 | |
| FGF5 | 2.07 (0.23) | 2.04 (0.33) | 0.03 |  |  |  | | | |  | |  | |
| IL10RA**^b^** | 2.26 (0.51) | 2.23 0.69) | 0.02 |  |  |  | | | |  | |  | |
| MCP1 | 13.19 (0.42) | 13.16 (0.57) | 0.02 |  |  | 12.00 (1.70) | | | | 11.76 (1.24) | | 0.24 | |
| ARTN | 1.77 (0.75) | 1.77 (0.70) | 0.01 |  |  | 2.11 (0.89) | | | | 2.16 (0.84) | | -0.05 | |
| CD8A | 12.00 (0.54) | 12.00 (0.52) | 0.01 |  |  | 3.57 (1.29) | | | | 3.45 (1.27) | | 0.12 | |
| CSF1 | 10.97 (0.23) | 10.96 (0.18) | 0.00 |  |  | 9.86 (0.86) | | | | 9.94 (0.81) | | -0.08 | |
| SCF | 10.34 (0.31) | 10.33 (0.36) | 0.00 |  |  | 3.58 (0.55) | | | | 3.65 (0.71) | | -0.08 | |
| CCL3 | 6.18 (0.58) | 6.18 (0.60) | 0.00 |  |  | 4.8 (1.87) | | | | 5.28 (1.60) | | -0.48 | |
| IFNgamma | 7.46 (0.92) | 7.48 (1.40) | -0.01 |  |  | 6.21 (2.17) | | | | 6.40 (1.63) | | -0.19 | |
| IL17C**^b^** | 2.28 (0.44) | 2.29 (0.60) | -0.01 |  |  |  | | | |  | |  | |
| IL18R1 | 9.52 (0.39) | 9.53 (0.39) | -0.01 |  |  | 9.22 (0.87) | | | | 9.20 (0.89) | | 0.01 | |
| LAPTGFbeta1 | 9.30 (0.37) | 9.31 (0.35) | -0.01 |  |  | 5.91 (0.94) | | | | 6.18 (0.83) | | -0.27 | |
| OPG | 10.95 (0.24) | 10.96 (0.37) | -0.01 |  |  | 9.34 (1.12) | | | | 9.28 (1.09) | | 0.06 | |
| IL10RB | 7.02 (0.26) | 7.04 (0.27) | -0.02 |  |  | 4.35 (0.53) | | | | 4.32 (0.67) | | 0.04 | |
| uPA | 11.3 (0.30) | 11.32 (0.29) | -0.02 |  |  | 9.76 (1.25) | | | | 10.12 (1.06) | | -0.35 | |
| CXCL11 | 9.36 (0.79) | 9.39 (1.13) | -0.03 |  |  | 6.11 (2.22) | | | | 5.97 (1.85) | | 0.14 | |
| CXCL9 | 7.61 (0.89) | 7.64 (0.97) | -0.03 |  |  | 8.81 (1.73) | | | | 8.99 (1.29) | | -0.17 | |
| IL15RA | 2.78 (0.23) | 2.82 (0.26) | -0.04 |  |  | 1.87 (0.29) | | | | 1.81 (0.31) | | 0.06 | |
| CCL11 | 7.53 (0.46) | 7.57 (0.46) | -0.05 |  |  | 1.9 (0.52) | | | | 1.83 (0.46) | | 0.07 | |
| IL17A | 2.96 (0.73) | 3.01 (1.06) | -0.05 |  |  | 2.55 (1.02) | | | | 2.57 (1.35) | | -0.02 | |
| NT3**^b^** | 3.29 (0.44) | 3.34 (0.38) | -0.05 |  |  |  | | | |  | |  | |
| TRAIL | 9.44 (0.28) | 9.48 (0.29) | -0.05 |  |  | 10.39 (1.21) | | | | 10.20 (0.84) | | 0.20 | |
| LIFR | 4.88 (0.26) | 4.93 (0.19) | -0.06 |  |  | 4.98 (0.81) | | | | 4.98 (0.75) | | 0.00 | |
| PDL1 | 7.43 (0.29) | 7.52 (0.40) | -0.09 |  |  | 5.04 (0.83) | | | | 4.86 (1.03) | | 0.19 | |
| CD5 | 6.42 (0.28) | 6.53 (0.33) | -0.11 |  |  | 5.5 (0.95) | | | | 5.53 (1.03) | | -0.03 | |
| BetaNGF**^b^** | 1.92 (0.12) | 2.04 (0.27) | **-0.12** |  |  |  | | | |  | |  | |
| IL24**^b^** | 1.99 (0.62) | 2.11 (0.91) | -0.12 |  |  |  | | | |  | |  | |
| LIF | 1.40 (0.46) | 1.53 (1.08) | -0.13 |  |  | 3.75 (1.03) | | | | 3.89 (0.85) | | -0.14 | |
| HGF | 10.39 (0.33) | 10.52 (0.44) | -0.14 |  |  | 9.00 (1.00) | | | | 9.27 (1.07) | | -0.26 | |
| CX3CL1 | 6.91 (0.35) | 7.08 (0.40) | -0.16 |  |  | 6.64 (0.87) | | | | 6.56 (0.87) | | 0.08 | |
| CXCL10 | 9.95 (0.88) | 10.12 (0.88) | -0.16 |  |  | 8.8 (3.04) | | | | 8.91 (2.52) | | -0.12 | |
| MMP10 | 9.20 (0.56) | 9.41 (0.66) | -0.20 |  |  | 8.49 (1.42) | | | | 8.62 (1.47) | | -0.13 | |
| IL10 | 5.19 (0.55) | 5.41 (0.80) | -0.22 |  |  | 3.16 (1.10) | | | | 3.47 (1.00) | | -0.31 | |
| IL8 | 7.09 (0.44) | 7.33 (0.67) | -0.23 |  |  | 12.46 (1.24) | | | | 12.87 (0.96) | | -0.41 | |
| TNFSF14 | 6.97 (0.48) | 7.32 (0.59) | **-0.35** |  |  | 6.98 (1.37) | | | | 7.37 (1.21) | | -0.39 | |
| TGFalpha | 6.05 (0.61) | 6.54 (0.85) | **-0.49** |  |  | 5.90 (0.94) | | | | 6.02 (0.91) | | -0.12 | |
| IL13**^b^** | 1.64 (0.68) | 2.19 (1.84) | -0.54 |  |  |  | | | |  | |  | |
| OSM | 6.81 (0.83) | 7.52 (0.95) | **-0.72** |  |  | 8.81 (1.28) | | | | 9.21 (1.08) | | -0.39 | |
| IL1alpha**^c^** |  |  |  |  |  | 9.35 (1.21) | | | | 9.68 (0.83) | | -0.32 | |
| IL22 RA1**^c^** |  |  |  |  |  | 3.63 (0.99) | | | | 3.78 (1.07) | | -0.15 | |

SD = standard deviation. Diff. = difference. NPX = normalized protein expression, an arbitrary unit in a Log2 scale according to the Proseek multiplex proximity enhanced extension assay provided by Olink Proteomics. All the detected biomarkers are sorted according to the differences between mean NPX values in JIA compared to controls in serum sorted in descending order, with corresponding biomarker differences found in saliva. Of the 92 proteins included in the inflammation panel. n = 87 were detected in

serum and n = 73 in saliva. Missing samples: n = 1 serum sample missing from the controls. and n = 4 saliva samples missing from individuals with JIA. Positive differences in NPX values indicate higher and negative values indicate lower biomarker levels in JIA compared to controls.

^a^ Numbers in bold indicate a statistically significant difference between mean NPX values (p < 0.05). Welch’s t-test was performed for normally distributed and Mann-Whitney U-test for

skewed biomarker data.

^b^ Biomarker detected in serum but not in saliva (n = 16)

^c^ Biomarker detected in saliva but not in serum (n = 2)
