# Supplementary material for: Serum and salivary inflammatory biomarkers in juvenile idiopathic arthritis—an explorative cross-sectional study
Source: Pediatr Rheumatol Online J. 2024 Mar 9;22:36. doi: 10.1186/s12969-024-00972-6 (PMC10924355; doi:10.1186/s12969-024-00972-6)
Supplement: Supplementary file 4 — Additional file 4: Supplemental Table S4. Serum and salivary inflammation-related biomarkers in active versus inactive JIA. The table shows all the detected biomarkers in serum and saliva, with mean NPX values and differences between mean NPX values in active versus inactive JIA. [file 12969_2024_972_MOESM4_ESM.docx]

| **Supplemental Table S4**. Serum and salivary inflammation-related biomarkers in active versus inactive JIA | | | | | | | | |  | |  |
| --- | --- | --- | --- | --- | --- | --- | --- | --- | --- | --- | --- |
|  | |  | **SERUM** |  |  |  |  | **SALIVA** | |  | |
|  | | **Active** (n = 21) | **Inactive** (n = 21) |  |  |  | **Active** (n = 18) | **Inactive** (n = 20) | |  | |
| **Proteins** | | **Mean NPX** | **Mean NPX** | **Diff. mean NPX^a^** |  |  | **Mean NPX** | **Mean NPX** | | **Diff. mean NPX^a^** | |
|  |  | **(±SD)** | **(±SD)** |  |  |  | **(±SD)** | **(±SD)** | |  |  |
| TNF | | 6.05 (1.88) | 5.34 (1.19) | 0.71 |  |  | 2.43 (0.95) | 2.70 (0.99) | | -0.27 | |
| MMP1 | | 11.56 (0.91) | 10.87 (1.18) | **0.69** |  |  | 6.33 (1.29) | 6.64 (1.29) | | -0.31 | |
| CXCL11 | | 9.64 (0.93) | 9.08 (0.50) | **0.56** |  |  | 5.33 (1.69) | 6.81 (2.44) | | **-1.48** | |
| IL6 | | 4.51 (1.19) | 3.96 (0.98) | 0.55 |  |  | 6.11 (1.54) | 6.23 (1.66) | | -1.12 | |
| S100A12 | | 6.81 (0.93) | 6.29 (1.16) | 0.52 |  |  | 8.10 (0.84) | 7.98 (1.35) | | 0.12 | |
| TNFB | | 7.08 (1.21) | 6.58 (0.70) | 0.50 |  |  | 2.40 (0.86) | 2.92 (1.26) | | -0.52 | |
| CXCL9 | | 7.85 (1.00) | 7.36 (0.71) | 0.49 |  |  | 8.06 (1.22) | 9.49 (1.87) | | **-1.43** | |
| MCP3^b^ | | 3.73 (0.69) | 3.32 (0.50) | **0.40** |  |  |  |  | |  | |
| CXCL5 | | 13.75 (0.55) | 13.44 (0.72) | 0.31 |  |  | 10.57 (2.69) | 12.27 (1.97) | | **-1.70** | |
| IFNgamma | | 7.62 (1.02) | 7.31 (0.81) | 0.31 |  |  | 5.82 (2.10) | 6.57 (2.22) | | -0.75 | |
| CXCL10 | | 10.10 (1.06) | 9.81 (0.64) | 0.29 |  |  | 7.69 (2.76) | 9.79 (3.00) | | **-2.11** | |
| FGF19 | | 9.22 (0.94) | 8.94 (0.78) | 0.28 |  |  | 1.89 (0.55) | 1.95 (0.57) | | -0.06 | |
| OSM | | 6.95 (0.66) | 6.66 (0.96) | 0.28 |  |  | 8.74 (0.91) | 8.88 (1.55) | | -0.14 | |
| ARTN | | 1.91 (0.96) | 1.64 (0.42) | 0.27 |  |  | 1.77 (0.51) | 2.41 (1.05) | | **-0.63** | |
| IL13^b^ | | 1.78 (0.70) | 1.51 (0.65) | 0.27 |  |  |  |  | |  | |
| 4E-BP1 | | 8.40 (0.77) | 8.14 (0.87) | 0.26 |  |  | 6.97 (1.48) | 7.90 (1.73) | | -0.93 | |
| IL7 | | 5.99 (0.47) | 5.73 (0.52) | 0.26 |  |  | 4.51 (0.81) | 5.10 (0.93) | | **-0.59** | |
| MMP10 | | 9.33 (0.54) | 9.07 (0.55) | 0.26 |  |  | 8.19 (1.20) | 8.75 (1.57) | | -0.56 | |
| CCL20 | | 7.66 (0.91) | 7.42 (0.76) | 0.23 |  |  | 6.12 (1.37) | 7.93 (2.48) | | **-1.81** | |
| IL17A | | 3.07 (0.83) | 2.84 (0.60) | 0.23 |  |  | 2.26 (0.94) | 2.82 (1.04) | | -0.56 | |
| IL17C^b^ | | 2.40 (0.46) | 2.17 (0.40) | 0.23 |  |  |  |  | |  | |
| STAMBP | | 5.45 (0.48) | 5.24 (0.53) | 0.22 |  |  | 4.95 (0.78) | 5.49 (1.30) | | -0.53 | |
| AXIN1 | | 3.19 (0.63) | 2.98 (0.46) | 0.21 |  |  | 1.58 (0.27) | 1.95 (0.75) | | -0.37 | |
| CDCP1 | | 3.83 (0.42) | 3.62 (0.29) | 0.21 |  |  | 7.77 (0.76) | 8.23 (1.11) | | -0.46 | |
| SLAMF1^b^ | | 2.75 (0.38) | 2.55 (0.31) | 0.20 |  |  |  |  | |  | |
| VEGFA | | 12.19 (0.66) | 11.99 (0.42) | 0.20 |  |  | 13.79 (0.54) | 14.14 (0.76) | | -0.35 | |
| CASP8 | | 2.75 (0.45) | 2.55 (0.45) | 0.19 |  |  | 5.84 (0.74) | 6.22 (1.49) | | -0.38 | |
| IL18 | | 9.60 (0.38) | 9.41 (0.33) | 0.19 |  |  | 9.54 (1.08) | 10.33 (1.35) | | -0.79 | |
| IL4^b^ | | 2.25 (1.37) | 2.06 (0.73) | 0.19 |  |  |  |  | |  | |
| NRTN^b^ | | 1.74 (0.70) | 1.55 (0.47) | 0.19 |  |  |  |  | |  | |
| CCL23 | | 10.83 (0.43) | 10.65 (0.35) | 0.18 |  |  | 2.09 (0.47) | 2.72 (1.08) | | **-0.62** | |
| IL18R1 | | 9.61 (0.38) | 9.44 (0.39) | 0.17 |  |  | 8.96 (0.49) | 9.45 (1.06) | | -0.50 | |
| ST1A1 | | 4.46 (0.76) | 4.29 (0.73) | 0.17 |  |  | 4.74 (1.06) | 5.11 (1.64) | | -0.37 | |
| CCL3 | | 6.26 (0.64) | 6.10 (0.52) | 0.16 |  |  | 4.38 (1.41) | 5.18 (2.17) | | -0.80 | |
| CSF1 | | 11.04 (2.24) | 10.89 (0.19) | **0.15** |  |  | 9.67 (0.87) | 10.03 (0.84) | | -0.35 | |
| CXCL1 | | 10.69 (0.44) | 10.54 (0.41) | 0.14 |  |  | 10.41 (1.60) | 11.49 (2.21) | | -1.08 | |
| FGF21^b^ | | 4.92 (1.27) | 4.78 (1.47) | 0.14 |  |  |  |  | |  | |
| IL10 | | 5.26 (0.60) | 5.12 (0.51) | 0.14 |  |  | 3.04 (0.96) | 3.26 (1.22) | | -0.23 | |
| CD244 | | 8.24 (0.33) | 8.11 (0.27) | 0.13 |  |  | 2.09 (0.42) | 2.46 (0.79) | | -0.38 | |
| HGF | | 10.45 (0.32) | 10.33 (0.33) | 0.13 |  |  | 8.97 (0.63) | 9.03 (1.27) | | -0.06 | |
| PDL1 | | 7.50 (0.34) | 7.37 (0.20) | 0.13 |  |  | 4.85 (0.74) | 5.22 (0.87) | | -0.37 | |
| SIRT2 | | 4.31 (0.75) | 4.18 (0.70) | 0.13 |  |  | 3.00 (0.57) | 3.39 (1.25) | | -0.40 | |
| CCL25 | | 7.04 (0.47) | 6.93 (0.39) | 0.11 |  |  | 1.42 (0.21) | 1.49 (0.21) | | -0.07 | |
| CCL19 | | 10.69 (0.88) | 10.59 (0.63) | 0.10 |  |  | 2.66 (1.58) | 4.28 (2.25) | | **-1.62** | |
| CCL28 | | 2.73 (0.52) | 2.62 (0.39) | 0.10 |  |  | 7.33 (0.92) | 8.30 (1.35) | | **-0.97** | |
| CXCL6 | | 11.00 (0.56) | 10.91 (0.59) | 0.09 |  |  | 7.18 (1.48) | 8.77 (2.27) | | **-1.58** | |
| FGF5 | | 2.12 (0.23) | 2.02 (0.22) | 0.09 |  |  |  |  | |  | |
| IL20^b^ | | 1.50 (0.16) | 1.41 (0.12) | **0.09** |  |  |  |  | |  | |
| CCL11 | | 7.56 (0.52) | 7.49 (0.40) | 0.07 |  |  | 1.72 (0.32) | 2.05 (0.62) | | **-0.34** | |
| FGF23 | | 3.00 (0.77) | 2.93 (0.33) | 0.07 |  |  | 1.84 (0.76) | 1.76 (0.37) | | 0.08 | |
| BetaNGF^b^ | | 1.95 (0.11) | 1.89 (0.12) | 0.06 |  |  |  |  | |  | |
| CD5 | | 6.45 (0.29) | 6.40 (0.27) | 0.05 |  |  | 5.20 (0.64) | 5.77 (1.11) | | -0.56 | |
| IL12B | | 7.70 (0.53) | 7.65 (0.42) | 0.05 |  |  | 2.80 (0.93) | 3.38 (0.88) | | -0.58 | |
| IL24^b^ | | 2.02 (0.64) | 1.97 (0.60) | 0.05 |  |  |  |  | |  | |
| LAPTGFbeta1 | | 9.33 (0.40) | 9.28 (0.35) | 0.05 |  |  | 5.83 (0.59) | 5.98 (1.19) | | -0.14 | |
| OPG | | 10.98 (0.23) | 10.93 (0.26) | 0.05 |  |  | 8.93 (0.76) | 9.72 (1.27) | | **-0.79** | |
| TWEAK | | 11.41 (0.29) | 11.36 (0.32) | 0.05 |  |  | 7.62 (0.96) | 8.19 (1.78) | | -0.57 | |
| IL2RB^b^ | | 2.32 (0.30) | 2.30 (0.34) | 0.02 |  |  |  |  | |  | |
| CD6 | | 6.77 (0.35) | 6.76 (0.40) | 0.01 |  |  | 1.80 (0.79) | 2.24 (1.06) | | -0.43 | |
| DNER | | 10.27 (0.23) | 10.27 (0.28) | 0.00 |  |  | 11.93 (0.30) | 11.98 (0.20) | | -0.05 | |
| IL15RA | | 2.78 (0.22) | 2.78 (0.25) | 0.00 |  |  | 1.76 (0.26) | 1.98 (0.29) | | **-0.22** | |
| MCP1 | | 13.19 (0.40) | 13.18 (0.46) | 0.00 |  |  | 11.36 (1.84) | 12.58 (1.36) | | **-1.22** | |
| CD40 | | 12.54 (0.26) | 12.54 (0.33) | -0.01 |  |  | 11.65 (0.60) | 12.14 (0.90) | | -0.49 | |
| MCP2 | | 10.59 (0.46) | 10.60 (0.76) | -0.01 |  |  | 3.84 (1.30) | 4.17 (1.62) | | -0.33 | |
| CST5 | | 6.33 (0.46) | 6.35 (0.40) | -0.02 |  |  | 8.77 (0.92) | 8.47 (0.77) | | 0.31 | |
| LIFR | | 4.87 (0.29) | 4.89 (0.24) | -0.02 |  |  | 4.81 (0.70) | 5.14 (0.89) | | -0.33 | |
| CD8A | | 11.99 (0.59) | 12.02 (0.50) | -0.03 |  |  | 2.98 (0.81) | 4.10 (1.42) | | **-1.12** | |
| GDNF^b^ | | 3.11 (0.72) | 3.15 (0.48) | -0.04 |  |  |  |  | |  | |
| uPA | | 11.28 (0.29) | 11.32 (0.32) | -0.04 |  |  | 9.60 (0.72) | 9.90 (1.60) | | -0.30 | |
| TNFSF14 | | 8.83 (0.53) | 8.91 (0.45) | -0.05 |  |  | 6.76 (0.70) | 7.18 (1.78) | | -0.42 | |
| IL10RB | | 6.99 (0.29) | 7.05 (0.24) | -0.06 |  |  | 4.20 (0.46) | 4.49 (0.56) | | -0.29 | |
| TGFalpha | | 6.02 (0.56) | 6.08 (0.67) | -0.06 |  |  | 5.74 (0.67) | 6.05 (1.12) | | -0.31 | |
| TRAIL | | 9.40 (0.31) | 9.47 (0.26) | -0.07 |  |  | 10.04 (1.03) | 10.71 (1.30) | | -0.67 | |
| IL20RA | | 1.96 (0.31) | 2.05 (0.78) | -0.08 |  |  | 3.66 (1.13) | 4.50 (1.00) | | **-0.84** | |
| TNFRSF9 | | 6.95 (0.49) | 7.00 (0.49) | -0.08 |  |  | 4.90 (0.75) | 5.42 (1.09) | | -0.52 | |
| IL10RA^b^ | | 2.21 (0.55) | 2.30 (0.47) | -0.09 |  |  |  |  | |  | |
| LIF | | 1.35 (0.31) | 1.45 (0.58) | -0.11 |  |  | 3.57 (1.10) | 3.91 (0.97) | | -0.34 | |
| IL8 | | 7.03 (0.39) | 7.16 (0.49) | -0.13 |  |  | 12.29 (0.77) | 12.61 (1.55) | | -0.32 | |
| MCP4 | | 15.00 (0.67( | 15.13 (0.51) | -0.13 |  |  | 4.98 (1.36) | 6.09 (1.97) | | **-1.12** | |
| CCL4 | | 7.83 (0.69) | 7.97 (0.45) | -0.14 |  |  | 2.98 (1.05) | 3.86 (1.72) | | -0.88 | |
| SCF | | 10.26 (0.35) | 10.41 (0.26) | -0.14 |  |  | 3.39 (0.48) | 3.75 (0.57) | | -0.36 | |
| TRANCE | | 6.83 (0.57) | 6.97 (0.51) | -0.15 |  |  | 2.81 (1.02) | 3.09 (0.98) | | -0.29 | |
| CX3CL1 | | 6.83 (0.32) | 7.00 (0.36) | -0.18 |  |  | 6.32 (0.92) | 6.93 (0.72) | | **-0.61** | |
| Flt3L | | 10.03 (0.43) | 10.20 (0.37) | -0.18 |  |  | 4.04 (0.47) | 4.39 (0.96) | | -0.35 | |
| ADA | | 6.88 (0.46) | 7.09 (0.35) | -0.21 |  |  | 6.82 (0.73) | 6.93 (1.71) | | -0.11 | |
| NT3^b^ | | 3.15 (0.29) | 3.43 (0.52) | -0.29 |  |  |  |  | |  | |
| IL5^b^ | | 1.95 (0.69) | 3.25 (2.39) | **-1.30** |  |  |  |  | |  | |
| IL-1alpha^c^ | |  |  |  |  |  | 9.36 (1.12) | 9.34 (1.32) | | 0.02 | |
| IL22 RA1^c^ | |  |  |  |  |  | 3.38 (0.86) | 3.85 (1.07) | | -0.48 | |
|  | SD = standard deviation, Diff. = difference, NPX = normalized protein expression, NPX = normalized protein expression, an arbitrary unit in a Log2 scale according to the Proseek multiplex proximity enhanced extension assay provided by Olink Proteomics. All the detected biomarkers are sorted according to the differences between mean NPX values in active compared to inactive JIA in serum sorted in descending order, with corresponding biomarker differences found in saliva. Of the 92 proteins included in the inflammation panel, n = 87 were detected in serum and n = 73 in saliva. Missing saliva samples: n = 4 samples in active, and n = 1 sample missing from inactive JIA.  ^a^ Numbers in bold indicate a statistically significant difference between mean NPX values (p < 0.05). Welch’s t-test was performed for normally distributed and the Mann-Whitney-U-test for skewed biomarker data. Positive differences in NPX values indicate higher and negative values lower biomarker levels in active compared to inactive JIA.  ^b^ Biomarker detected in serum but not in saliva (n = 16)  ^c^ Biomarker detected in saliva but not in serum (n = 2). | | | | | | | | | | |
|  |  | | | | | | | | | | |
|  |  | | | | | | | | |  | |
|  |  | | | | | | | | |  | |
|  |  | | | | | | | | |  | |
|  | | |  |  |  |  |  |  | |  | |
|  | | | |  |  |  |  |  | |  | |
|  | | | |  |  |  |  |  | |  | |
